# Supplementary material for: 4-1BB-Based CAR T Cells Effectively Reverse Exhaustion and Enhance the Anti-Tumor Immune Response through Autocrine PD-L1 scFv Antibody
Source: Int J Mol Sci. 2023 Feb 20;24(4):4197. doi: 10.3390/ijms24044197 (PMC9961031; doi:10.3390/ijms24044197)
Supplement: Supplementary file 1 [file ijms-24-04197-s001.zip › ijms-2101168-supplementary.pdf]

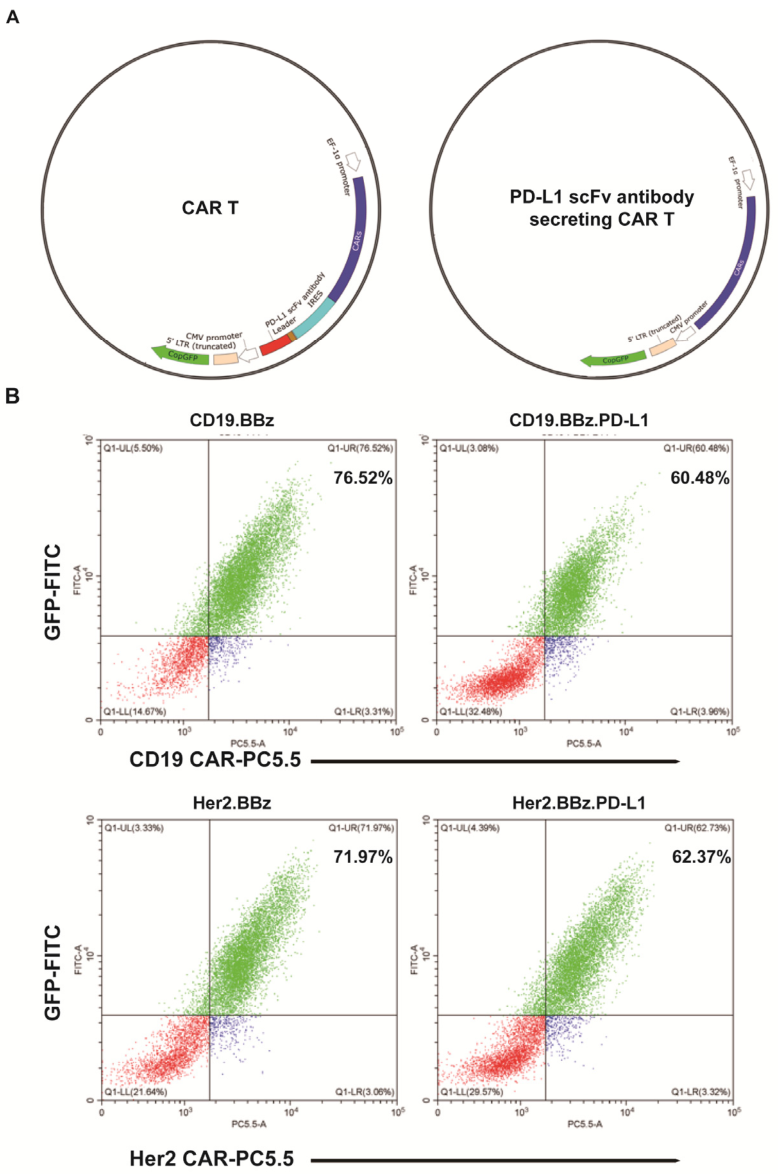

Figure S1. Generation of CAR T cells. (A) The plasmid profiles for CAR T and PD-L1 scFv antibody secreting CAR T. (B) The expression levels of CD19 and Her2 CAR were measured respectively along with GFP which detected by flow cytometry.

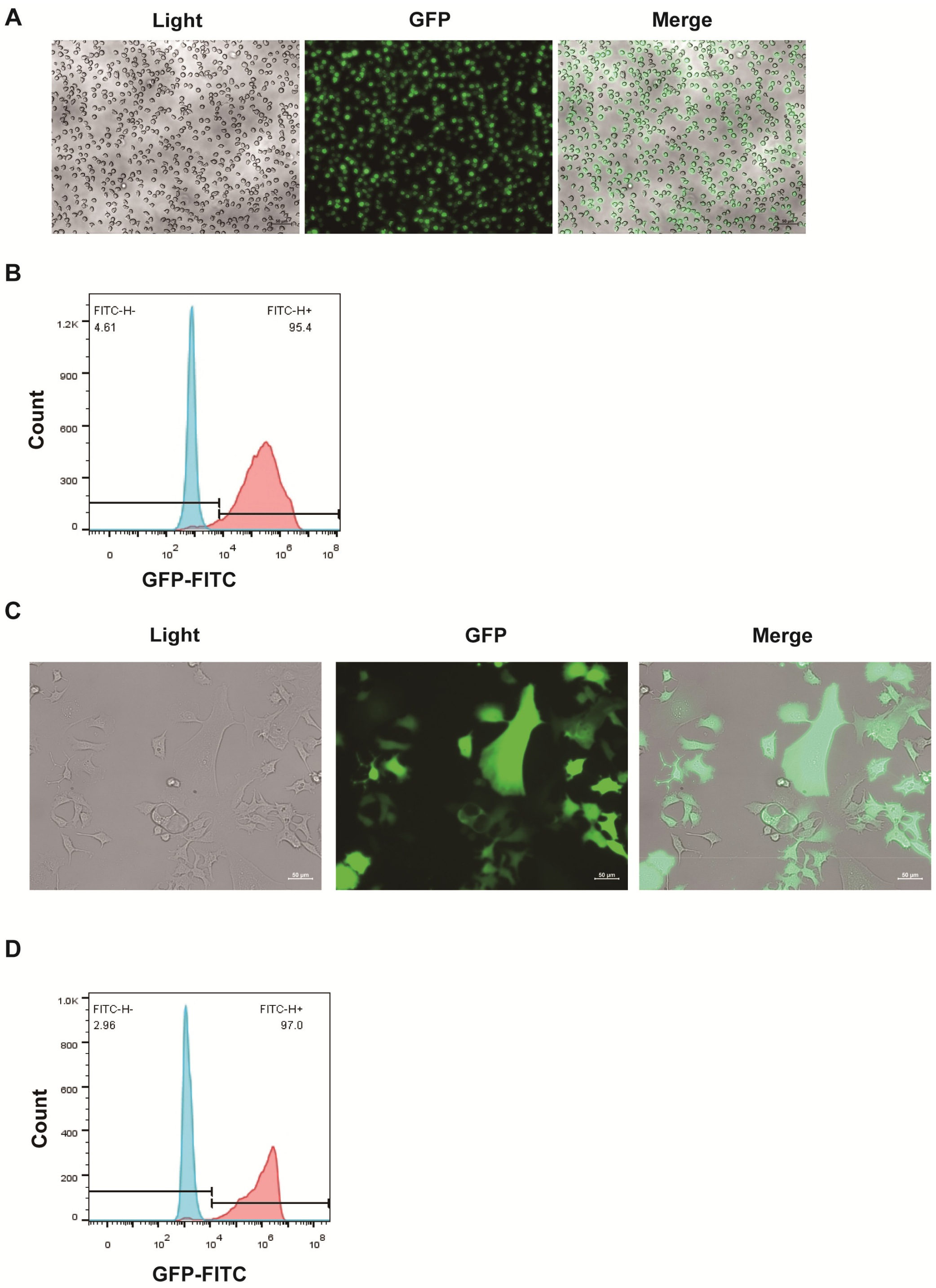

Figure S2. Establishment of Luc+Nalm6 cell line and Luc+HCC1954 cell line. (A) The GFP expression in Luc+Nalm6 cells was observed under fluorescence microscope. (B) The expression level of GFP in Luc+Nalm6 cells was detected by flow cytometry using parental Nalm6 cells as a control. (C) The GFP expression in Luc+HCC1954 cells was observed under fluorescence microscope. (D) The expression level of GFP in Luc+HCC1954 cells was detected by flow cytometry using parental Nalm6 cells as a control.

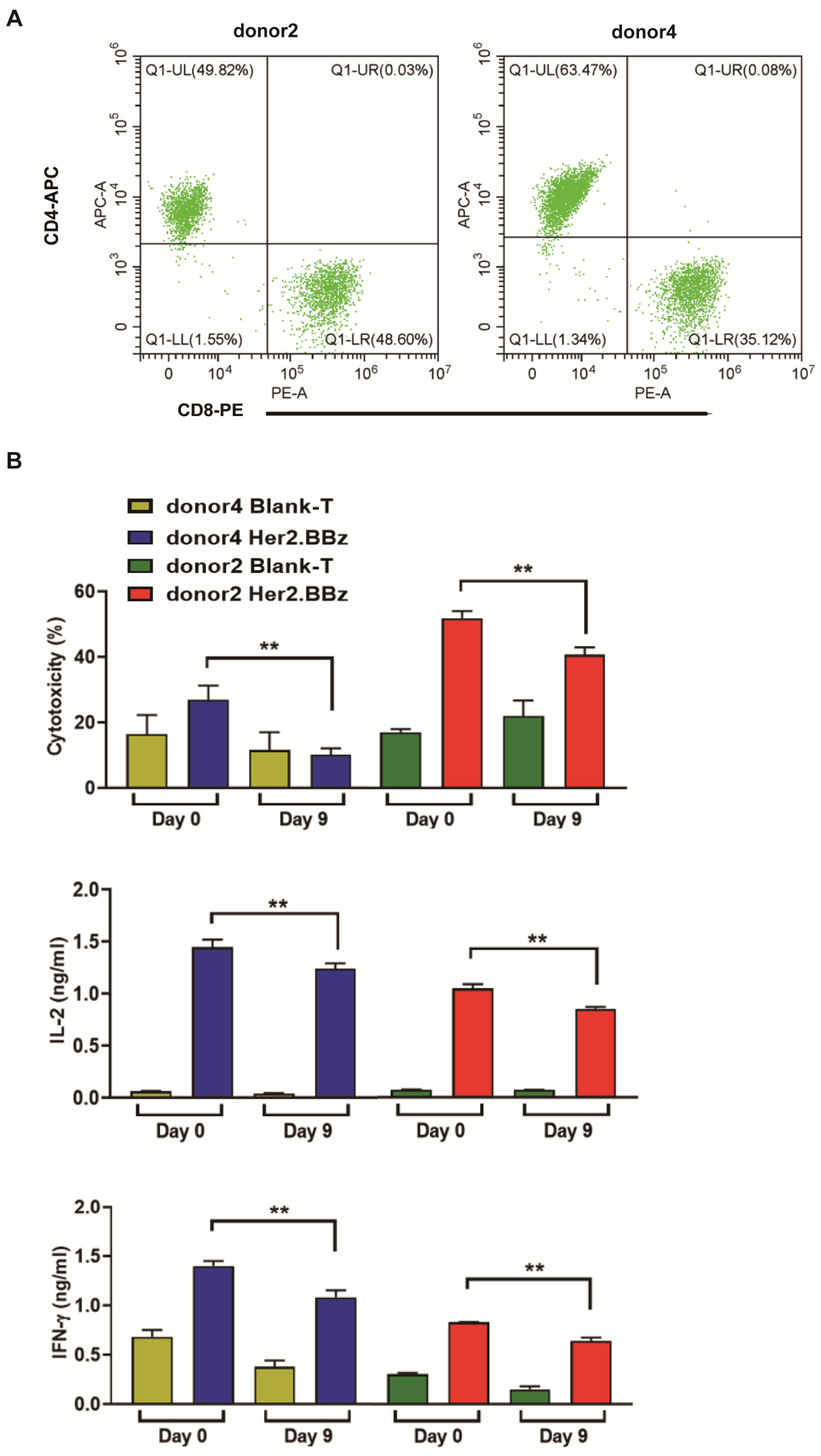

Figure S3. The compare of donor 2 and donor 4. (A) The proportions of CAR+CD4+ and CAR+CD8+ T cells were measured by flow cytometry. (B) The cytotoxicity and the cytokine secretion were measured before (day 0) and after (day 9) antigen stimulation at the E:T ratio of 1:10 (n=3).
